# Impact of maternal body mass index on pregnancy outcomes following frozen embryo transfer: A systematic review and meta-analysis

**DOI:** 10.1371/journal.pone.0319012

**Published:** 2025-03-21

**Authors:** Chucheng Tang, Fengming Tu

**Affiliations:** 1 Department of Reproductive, Huzhou Maternity & Child Health Care Hospital, Huzhou City, Zhejiang Province, China; 2 Department of Obstetrics, Huzhou Maternity & Child Health Care Hospital, Huzhou City, Zhejiang Province, China; King Saud University / Zagazig University, EGYPT

## Abstract

**Objective:**

There is still a significant gap in understanding how maternal body mass index (BMI) impacts outcomes of pregnancy after frozen embryo transfer (FET). This review aims to evaluate the effects of various BMI categories on clinical pregnancy and live birth rates in women undergoing FET.

**Methods:**

PubMed, Scopus, Embase, and Web of Science databases were searched for studies, published up to March, 2024, using the keywords “obesity”, “overweight”, “obese”, “maternal body mass index,” “pregnancy outcomes,” “frozen embryo transfer,”. Eligible studies were selected based on predefined inclusion criteria, statistical analysis was performed using a random-effects model, and ther results were presented as odds ratios (OR) with 95% confidence intervals (CI).

**Results:**

A total of 17 studies were included in the meta-analysis. Pooled findings indicate significantly reduced live birth rate in underweight (OR 0.93; 95% CI: 0.89, 0.98) and obese (OR 0.85; 95% CI: 0.77, 0.93) women but not in those who were overweight (OR 0.96; 95% CI: 0.92, 1.00), compared to those with normal BMI. Further, only those women who were underweight (OR 0.91; 95% CI: 0.85, 0.97) had reduced odds of clinical pregnancy rate but not those who were overweight (OR 0.99; 95% CI: 0.94, 1.05) or obese (OR 0.92; 95% CI: 0.82, 1.03).

**Conclusion:**

Maternal BMI impacts pregnancy outcomes after frozen embryo transfer, with underweight and obese women having lower live birth rates and only underweight women showing reduced clinical pregnancy rates compared to those with normal BMI. These findings underscore the importance of addressing BMI in women undergoing FET to improve pregnancy outcomes.

## Introduction

In recent years, there has been a growing interest in understanding the intricate relationship between maternal body mass index (BMI) and pregnancy outcomes, particularly in the context of assisted reproduction technology (ART) [1,2]. Excessive weight and obesity have been consistently linked with various adverse reproductive outcomes, ranging from disruptions in hormonal balance and ovulation to compromised embryo implantation and increased risks of pregnancy complications such as miscarriage and preeclampsia [3–6]. Conversely, low BMI may also affect fertility and pregnancy outcomes by disrupting hormonal equilibrium and impairing reproductive function, albeit through different mechanisms [7–9]. For women undergoing ART procedures, elevated BMI presents additional challenges, including the need for higher doses of fertility medications, an increased likelihood of ovarian hyperstimulation syndrome, higher rates of miscarriage, and lower rates of successful embryo implantation [10,11]. These challenges can contribute to significant emotional and financial burden on patients and on couples seeking fertility treatments [12,13].

Currently, frozen embryo transfer (FET) has emerged as a promising alternative to traditional fresh embryo transfer [14,15]. FET offers several advantages, such as the ability to better synchronize embryo transfer with the optimal uterine environment and increased flexibility in treatment scheduling [16,17]. Understanding the mechanisms through which maternal BMI may influence pregnancy outcomes following FET is crucial for improving reproductive success in patients undergoing ART [18,19]. However, the impact of maternal BMI on pregnancy outcomes following FET is still unclear [17,20]. Moreover, the existing literature often lacks comprehensive classification of outcomes based on BMI categories [17], particularly distinguishing between underweight, normal-weight, overweight, and obese patients. Therefore, it is challenging to establish specific effects of BMI on pregnancy outcomes in FET cycles [21,22], which prevents the clinicians from developing tailored treatment strategies.

This systematic review aims to address the existing gaps in the literature by evaluating the impact of BMI on specific pregnancy outcomes, including live birth rate and clinical pregnancy rate, stratified by BMI categories.

## Methods

The aims and methods of this meta-analysis have been registered with the International Prospective Register for Systematic Reviews, with ID number CRD42024528123 (available from https://www.crd.york.ac.uk/PROSPERO). The meta-analysis was conducted using the guidelines and checklist outlined by the Preferred Reporting Items for Systematic reviews and Meta-Analyses (PRISMA) Group (S1 File).

### Literature search

PubMed, Scopus, Embase, and Web of Science databases were systematically searched for studies, published from inception of the databases up to March 31^st^ 2024. We used the following Medical Subject Heading and free words to determine the search strategy: “obesity”, “overweight”, “obese”, “body mass index,” “pregnancy,” “frozen”, “frozen embryo transfer”, “blastocyst transfer”, “assisted reproductive technology” “and “birth rate”. Further details are presented in S1 Table. We also explored reference lists in retrieved studies and prior systematic reviews for potentially relevant studies. Two reviewers conducted the search in all databases independently. They collated all search results and deduplicated them using EndNote. The remaining unique studies underwent further screening, first by title/abstract and then by full-texts to include relevant articles in the review. Disagreements between reviewers were resolved by discussion.

### Eligibility criteria

Inclusion criteria was determined as follows: 1) Study population: adult females undergoing FET 2) Exposure group: Obese, overweight, or underweight females as determined by BMI 3) Comparator group: Normal BMI females 4) Outcomes: clinical pregnancy and live birth rates 5) Study design: All types of comparative studies. 5) Studies reporting confounder adjusted effect sizes for the outcomes of interest.

Exclusion criteria was as follows: 1) Studies on other ART modalities 2) Studies not segregating data based on BMI 3) Studies not reporting required outcomes or reporting unadjusted outcomes 4) Abstracts, unpublished data, theses, reviews and editorials.

### Quality assessment and data management

Quality of the cohort studies was assessed using the ROBINS-I instrument. Two reviewers independently scrutinized the methodological quality of the included studies and any disagreements were resolved by consensus.

Data were extracted from the relevant studies, and included study name, location, design, sample size, age distribution, BMI categories (underweight, normal weight, overweight, and obese), duration of infertility (in years), and outcomes of interest (i.e., live births and clinical pregnancies) based on BMI categories.

### Statistical analysis

STATA version 15.0 was used for the analysis. We adopted a random-effects model to assess the relationship between maternal BMI categories, usually defined as underweight (BMI <  18.5 kg/m^2^), overweight (BMI 25-29.9 kg/m^2^), and obese (BMI ≥  30 kg/m^2^), and pregnancy outcomes following FET. Specifically, we assessed the rate of live births and clinical pregnancy across different BMI categories compared to healthy normal-BMI women. Heterogeneity among the studies was evaluated by I^2^ statistics [23]. Forest plot and Egger’s test was used to assess the publication bias [24]. A p <  0.05 was used as the threshold for statistical significance.

## Results

### General characteristics of included studies

**Fig 1:**
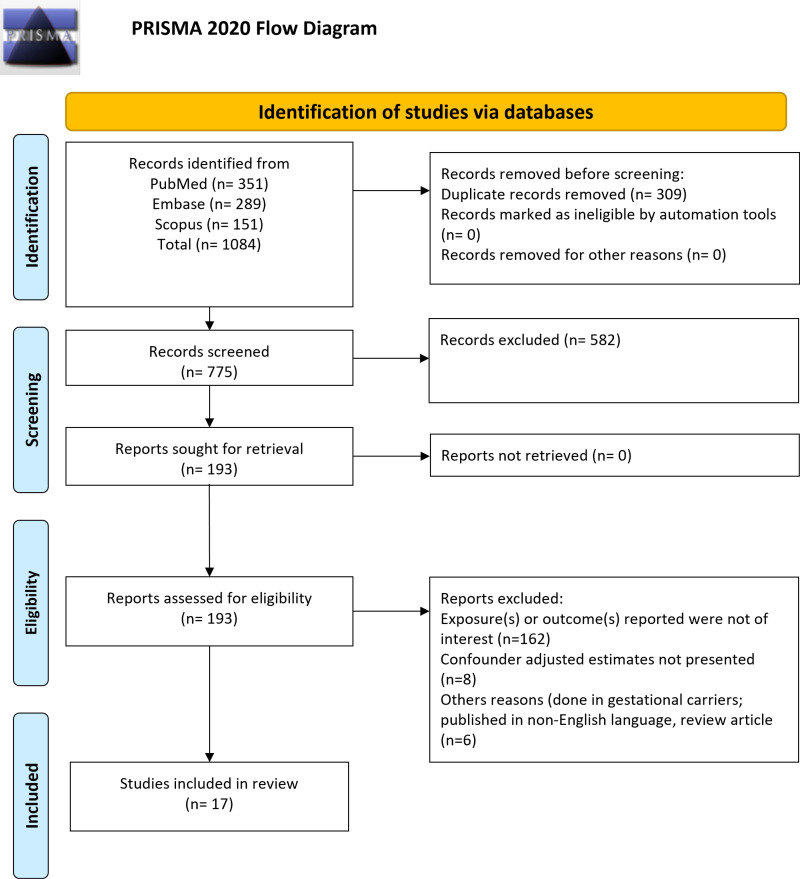
Flow diagram of literature screening.

A total of 1084 studies were identified by the literature search. Of them, 309 studies were removed as duplicates. Of the remaining 775 studies, 582 were eliminated at the stage of the title and abstract review. Full texts of the 193 studies were assessed for eligibility. Finally, 17 studies were included in the analysis [7,19,25–39] (Fig 1, S2 File).

As summarized in Table 1, all the included studies had a retrospective cohort design. The detailed data extracted from these studies are presented in Table 1.

**Table 1:** Details of included studies.

References; study design	Country	Groups	Sample size	Mean age (years)	BMI definition (kg/m^2^)	Infertility duration (years)	Adjustment done for
Beshar et al. (2023) [25] RC	USA	Normal Overweight Obese	229 128 68	22 26.3 33.0	18.5-24.9 25-30 >30	NR	Age at transfer, nulliparity, embryo grade, race/ethnicity, endometrial thickness on day of hCG trigger, and diagnosis of unexplained infertility
Bakkensen et al. (2024) [26] RC	USA	Underweight Normal Overweight Obese	1612 31666 13419 9867	34.3 ± 4.4 35.2 ± 4.1 35.4 ± 4.1 35.6 ± 4.1	<18.5 18.5-24.9 25-29.9 ≥30	NR	Age at transfer, race and ethnicity, prior pregnancy loss, current smoking, indication for preimplantation genetic testing, and endometrial thickness
Peterson et al. (2024) [27] RC	USA	Underweight Normal Overweight Obese	1734 33126 13068 7960	34.7 ± 4.0 35.1 ± 4.2 35.3 ± 4.2 35.6 ± 4.0	<18.5 18.5-24.9 25-29.9 >30	NR	Age, cycle order, race, male factor infertility, and female factor infertility
Liu and Shi (2024) [28] RC	China	Underweight Normal Overweight Obese	81 637 108 25	30.6 ± 4.0 31.1 ± 3.4 31.7 ± 4 30 ± 4.2	<18.5 18.5-24.9 25-30 >30	2.2 ± 2.1 2.0 ± 2.1 2.8 ± 2.5 3.0 ± 2.0	Infertility duration, endometrial thickness, infertility type (primary infertility vs secondary infertility), protocol in fresh cycle (agonist, antagonist, other), biopsied blastocysts, no result embryos
Fawarseh et al. (2022) [29] RC	Israel	Underweight Normal Overweight Obese	43 286 154 158	33.3 ± 7.2 34.9 ± 6.2 35.9 ± 7.4 36.1 ± 5.2	<18.5 18.5-24.9 25-30 >30	NR	Maternal age, endometrial thickness, and KID scores (reflecting embryo quality)
Shen et al. (2022) [30] RC	China	Underweight Normal Overweight Obese	2422 13845 6037 1178	31 ± 4.3 32.3 ± 4.8 33.1 ± 5.2 31.9 ± 4.9	<18.5 18.5- < 23 23- < 27.5 ≥27.5	3.1 ± 2.6 3.2 ± 2.9 3.5 ± 3.3 3.7 ± 3.3	Adjustment for confounders done; however, variables adjusted were not mentioned
Kidera et al. (2023) [31] RC	Japan	Underweight Normal Overweight Obese	943 3814 935 330	38 39 40 41	<18.5 18.5-22.5 22.5 to 25 >25	NR	Propensity score matched
Zheng et al. (2022) [32] RC	China	Underweight Normal Overweight Obese	1127 6925 1810 390	30.2 ± 3.9 31.9 ± 4.5 32.8 ± 5.2 31.7 ± 4.6	<18.5 18.5-24 24 to 28 ≥28	3.0 ± 1.9 3.2 ± 2.3 3.6 ± 2.6 3.8 ± 2.4	Maternal age, type of infertility, IVF indications, antral follicle count (AFC), endometrial thickness, type of endometrial preparation, expansion stage, inner cell mass, and trophectoderm
Zeng et al. (2023) [33] RC	China	Underweight Normal Overweight Obese	2136 11723 2622 495	NR	<18.5 18.5-23.9 24 to 27.9 ≥28	NR	Maternal age, paternal age, causes of infertility, protocol, number of high-quality embryos and D3/D5 transferred embryos
Hu et al. (2024) [34] RC	China	Underweight Normal Overweight Obese	137 1130 339 61	28 29 29 29	<18.5 18.5- < 25 25 to < 30 ≥30	3 3 3 4	Maternal age, number of embryos transferred, stage of embryo development, endometrial preparation protocol, fertilization method, cause of infertility, endometrial thickness, and number of oocytes retrieved
Insogna et al. (2017) [35] RC	USA	Underweight Normal Overweight Obese	8 288 106 59	34.8 ± 3.2 35.4 ± 4.2 36.6 ± 5.0 36.6 ± 3.9	<18.5 18.5-24.9 25 to 29.9 ≥30	NR	Cohort score (marker of embryo quality), uterine cause of infertility, mock transfer score, maternal age, transfer of more than one embryo, diminished ovarian reserve, and male factor infertility
Zhang et al. (2019) [19] RC	China	Underweight Normal Overweight Obese	2527 13224 5079 1213	30.5 31.3 31.6 31.1	<18.5 18.5- < 23 23 to 27.5 >27.5	3.2 ± 2.3 3.3 ± 2.7 3.6 ± 2.9 4.1 ± 2.9	Maternal age, infertility duration, gravidity, parity, main cause of infertility, number of OPU prior to FET, year of treatment, number of embryos transferred, and embryo developmental stage at transfer
Lin et al. (2019) [36] RC	China	Underweight Normal Obese	972 480 228	32.8 ± 3.4 33.1 ± 3.8 33.3 ± 3.6	18.5-24.9 25 to 29.9 ≥30	3.8 ± 2.7 42.9 4.2 ± 2.9	Maternal age, infertility duration, duration of cryopreservation, endometrial thickness, embryo quality, means of preparing the endometrium, number of embryos transferred as well as embryo developmental stage
Prost et al. (2020) [37] RC	France	Normal Obese	799 159	32.9 ± 4.1 32.9 ± 4.8	18.5- 24.9 ≥30	NR	Maternal age, smoking status, serum AMH, endometrium thickness, parity, infertility cause, double blastocyst transfer
Qiu et al. (2019) [7] RC	China	Underweight Normal Overweight Obese	184 1911 780 204	28.9 ± 3.2 29.9 ± 3.4 30.5 ± 3.9 30 ± 3.7	<18.5 18.5-24.9 25 to 29.9 ≥30	3.1 ± 2.0 3.5 ± 2.6 3.9 ± 2.8 4.2 ± 2.9	Female age, duration of infertility, gravidity, parity, history of preterm delivery, indication combined with PCOS, previous IVF failures, AFC, fertilization methods, embryo stage at transfer, endometrial thickness on ET day, endometrial preparation, and the number of embryos transferred
Tang et al. (2021) [38] RC	China	Underweight Normal Overweight	1315 6230 1210	33.6 33.8 33.7	<18.5 18.5-24.9 ≥25	NR	Age of embryo transfer, age of oocyte retrieval, infertility duration, endometrial thickness, embryo quality, number of embryos transferred, and embryo developmental stage
Oliva et al. (2021) [39] RC	USA	Underweight Normal	314 4420	NR	<18.5 18.5-24.9	NR	Maternal age, markers of ovarian reserve, total gonadotropin dose, stimulation type, trigger type, estradiol and progesterone at the time of surge, number of embryos transferred, day of embryo transfer, endometrial type, and morphologic grade

RC, retrospective cohort; NR, not reported; BMI, body mass index; PCOS, polycystic ovary syndrome.

### Participant information

The analysis included information of 237,562 women, with the mean age of around 32 years. Based on the BMI, women were categorized as underweight (n = 15,272), normal-weight (n = 130,733), overweight (n-46,767), and obese (n = 22,395).

### Assessment of study quality

Quality of the cohort studies was evaluated using the ROBINS-I tool, detailed by Sterne et al. (2016). As summarized in Table 2, most studies had a high risk of bias. Additionally, several studies had missing data and indications of selection bias (Table 2).

**Table 2:** Bias risk assessment for inclusion in the study.

References	Confounding	Selection	Deviation	Missing data	Measurement of outcomes	Reporting	Classification	Risk of bias
Beshar et al. (2023) [25]	+	+	–	+	+	?	+	Some concerns
Bakkensen et al. (2024) [26]	+	?	?	+	+	+	+	Some concerns
Peterson et al. (2024) [27]	–	+	+	+	+	+	+	Low
Liu and Shi (2024) [28]	+	+	+	+	–	?	+	Some concerns
Fawarseh et al. (2022) [29]	+	+	+	+	+	–	+	Low
Shen et al. (2022) [30]	+	+	–	+	+	?	+	Some concerns
Kidera et al. (2023) [31]	+	?	?	+	+	+	+	Some concerns
Zheng et al. (2022) [32]	+	?	?	+	+	+	+	Some concerns
Zeng et al. (2023) [33]	+	?	+	+	+	+	+	Some concerns
Hu et al. (2024) [34]	–	+	–	+	–	?	?	Very low
Insogna et al. (2017) [35]	+	?	?	+	–	+	+	Some concerns
Zhang et al. (2019) [19]	–	+	–	+	+	?	+	Low
Lin et al. (2019) [36]	+	?	?	+	–	+	+	Low
Prost et al. (2020) [37]	+	?	?	+	+	+	+	Some concerns
Qiu et al. (2019) [7]	+	+	–	+	+	?	+	Some concerns
Tang et al. (2021) [38]	+	?	?	+	–	+	+	Low
Oliva et al. (2021) [39]	+	?	?	+	+	+	+	Some concerns

### Meta-analysis outcome

#### Live birth rate.

**Fig 2:**
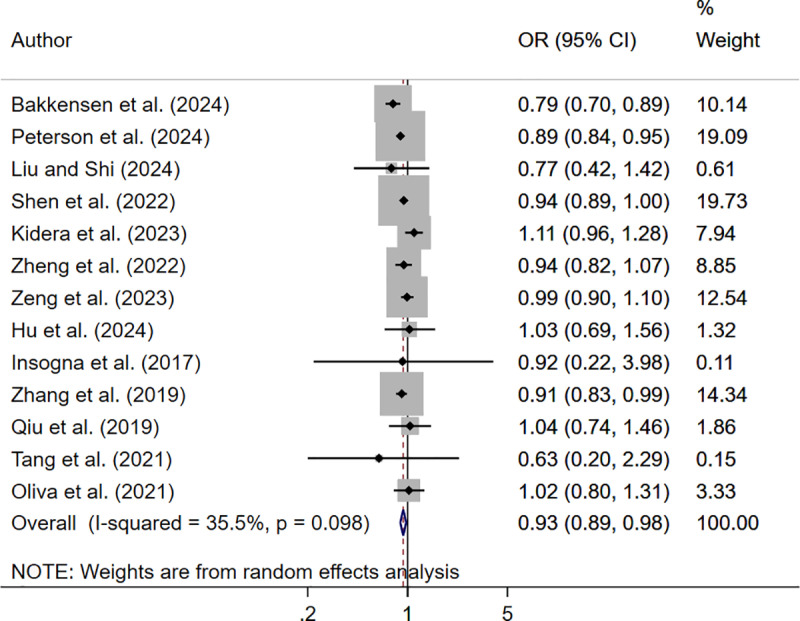
Forest plot comparing live birth rate in underweight and normal-BMI women.

**Fig 3:**
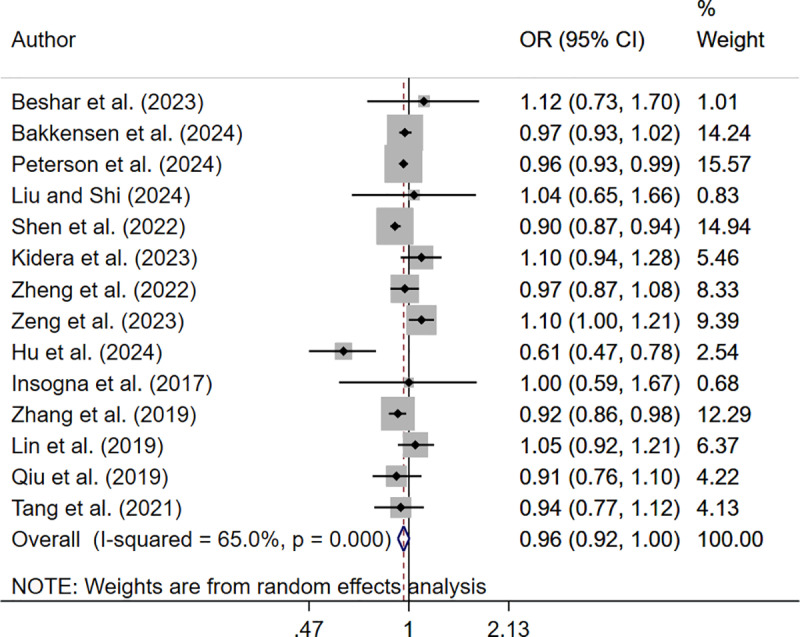
Forest plot comparing live birth rate in overweight and normal-BMI women.

**Fig 4:**
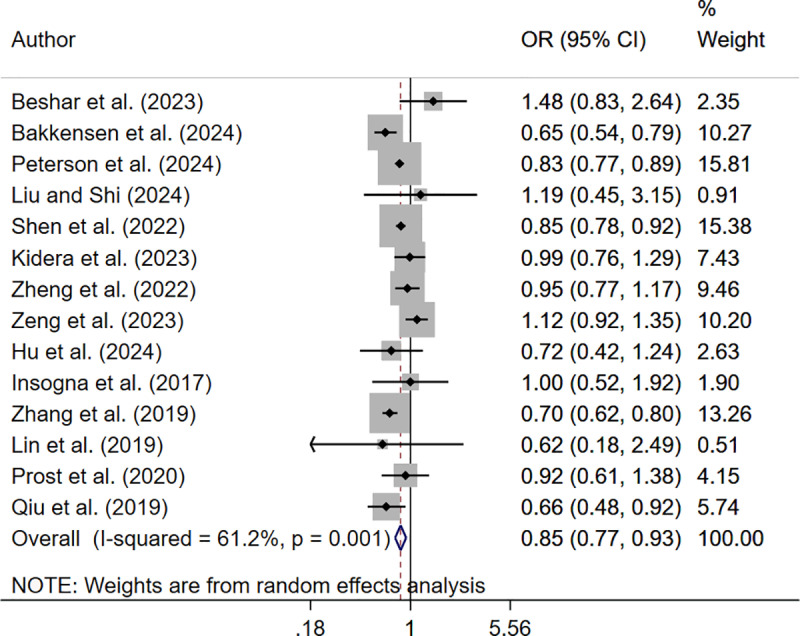
Forest plot comparing live birth rate in obese and normal-BMI women.

The analysis reported significantly reduced reduced live birth rate in underweight (OR 0.93; 95% CI: 0.89, 0.98, I^2^ = 35.5%, N = 13) and obese (OR 0.85; 95% CI: 0.77, 0.93, I^2^ = 61.2%, N = 14) women but not in those who were overweight (OR 0.96; 95% CI: 0.92, 1.00, I^2^ = 65.0%, N = 14), compared to those with normal BMI (Figs 2–4).

There was no evidence of publication bias for comparison of live birth in any of the three BMI categories (underweight, Egger’s p = 0.71; overweight, p = 0.66 and obese, p = 0.56). The funnel plots also support lack of potential publication bias (S1–S3 Figs).

#### Clinical pregnancy rate.

**Fig 5:**
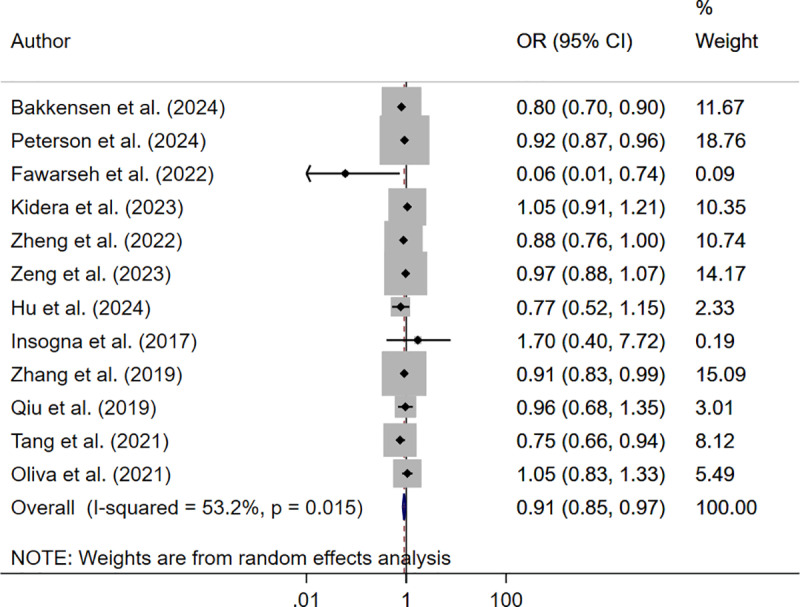
Forest plot comparing clinical pregnancy rate in underweight and normal-BMI women.

**Fig 6:**
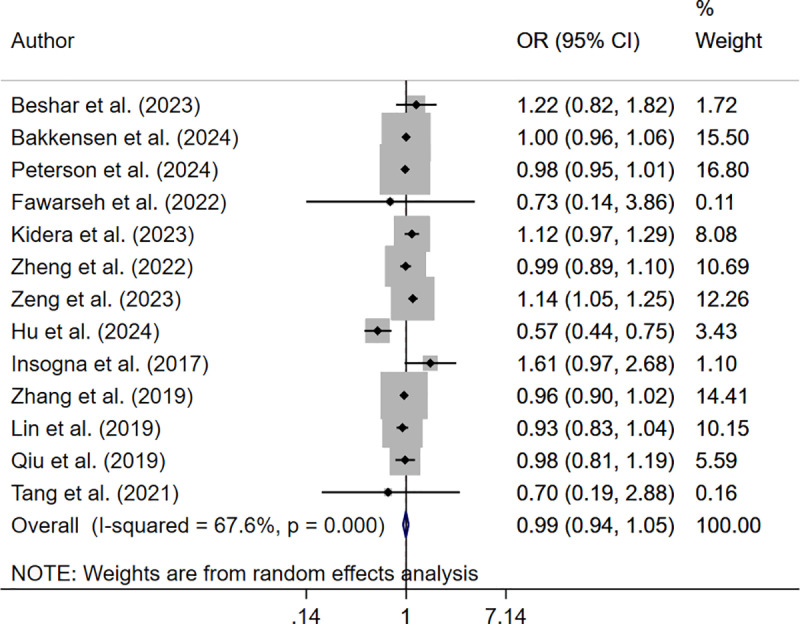
Forest plot comparing clinical pregnancy rate in overweight and normal-BMI women.

**Fig 7:**
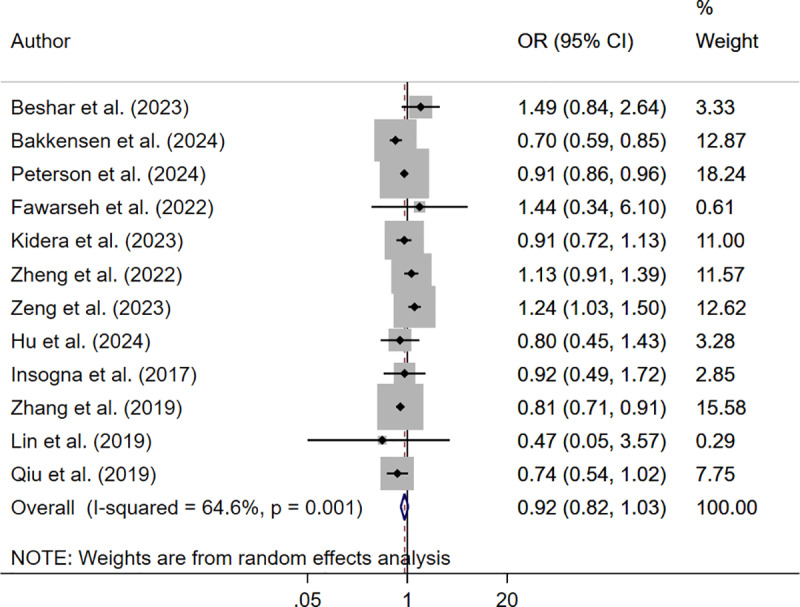
Forest plot comparing clinical pregnancy rate in obese and normal-BMI women.

The pooled analysis showed that only those women who were underweight (OR 0.91; 95% CI: 0.85, 0.97, I^2^ = 53.2%, N = 12) had reduced odds of clinical pregnancy rate. Those who were overweight (OR 0.99; 95% CI: 0.94, 1.05, I^2^ = 67.6%, N = 13) or obese (OR 0.92; 95% CI: 0.82, 1.03, I^2^ = 64.6%, N = 12) had similar clinical pregnancy rate compared to women with normal BMI (Figs 5–7).

There was no evidence of publication bias for comparison of live birth in any of the three BMI categories (underweight, Egger’s p = 0.43; overweight, p = 0.92 and obese, p = 0.79). The funnel plots also support lack of potential publication bias (S4–S6 Figs).

## Discussion

Our systematic review and meta-analysis provides evidence of the association between the maternal body weight and FET outcomes.

### Live birth rates

Our meta-analysis revealed a significant association between abnormal maternal BMI and live birth rates for underweight and obese women but not for those who were overweight. These findings underscore the importance of considering maternal BMI as a critical determinant of pregnancy success in FET procedures. Firstly, the observed reduction in live birth rates among underweight women highlights the potential adverse effects of insufficient maternal weight on embryo implantation and subsequent pregnancy maintenance [40]. Underweight women may experience hormonal imbalances and inadequate uterine receptivity, impairing embryo implantation and leading to lower live birth rates [41].

Conversely, significant reduction in live birth rates in obese women underscores the detrimental effects of excessive maternal weight on fertility outcomes post-FET [42]. Elevated BMI levels are associated with various metabolic and endocrine dysregulations, including insulin resistance and altered hormonal profiles, which can negatively impact oocyte quality, embryo development, and implantation [32,43]. Moreover, excessive increase in maternal adiposity may contribute to chronic inflammation and oxidative stress, further compromising reproductive outcomes [44,45]. Overweight women might not reach the threshold of physiological disturbances seen in obesity, such as hormonal imbalances and inflammatory responses, which can adversely affect implantation and pregnancy maintenance. Additionally, variations in study populations and methodologies might have contributed to the observed lack of association.

### Clinical pregnancy rate

Our results showed, that being underweight was associated with decreased clinical pregnancy rates, compared to those of the normal-weight group. However, clinical pregnancy rate in the obese and overweight group was comparable to that of the normal-weight group. We may speculate that while obesity is widely recognized as a risk factor for adverse pregnancy outcomes, the impact of being obese on fertility is less clear-cut. Similar clinical pregnancy rate in overweight and obese women, compared to normal BMI women, might be linked to the presence of adipose tissue, which could provide a more favorable hormonal and metabolic milieu for embryo implantation and development [7,46–48]. Additionally, increased availability of energy reserves in these women might have a supportive effect [49,50]. However, these studies caution that despite a possible protective benefit of the overweight or obese status, a higher BMI might negatively impact pregnancy outcomes, as observed in our study. At the same time, reduced rate of clinical pregnancy in underweight women may stem from potential changes in hormonal profiles and reproductive function associated with low BMI [51,52]. Studies have shown that inadequate energy reserves in underweight patients might compromise follicular development and oocyte quality, thereby affecting the likelihood of successful implantation and clinical pregnancy [53,54].

Additionally, it’s crucial to consider the potential role of confounding variables and effect modifiers that may influence the association between maternal weight and live birth rates. Factors such as age, parity, underlying medical conditions, lifestyle factors, and access to healthcare services could confound or modify the observed relationship. For instance, older women, who are more likely to be overweight or obese, may also face age-related declines in fertility, which could attenuate the negative impact of excess weight on clinical pregnancy rates. Similarly, socioeconomic disparities and disparities in healthcare access could contribute to variations in pregnancy outcomes across different weight categories.

### Implications for clinical practice

The observed associations between maternal BMI and pregnancy outcomes carry implications for clinical practice. Rather than adopting a one-size-fits-all approach, personalized interventions that meticulously consider individual BMI profiles are essential. By tailoring treatment plans to accommodate the specific needs and challenges associated with varying BMI categories, clinicians can significantly enhance the likelihood of successful outcomes in fertility treatments. For instance, healthcare providers should prioritize pre-conception counseling and interventions aimed at optimizing maternal BMI to enhance the success of FET procedures [55]. In underweight women, nutritional supplementation and lifestyle modifications may improve fertility outcomes [56]. Similarly, overweight and obese women may benefit from weight management strategies, including diet modifications, physical activity interventions, and, in some cases, bariatric surgery, to mitigate the adverse effects of excess adiposity on fertility and pregnancy [57].

### Challenges and considerations in BMI stratification

While our meta-analysis offers valuable insights into the association between maternal BMI and FET outcomes, it is crucial to acknowledge the challenges inherent in BMI stratification that require careful consideration. One notable challenge is the lack of standardized classification of outcomes based on BMI categories across the existing literature. This deficiency affects our the ability to accurately evaluate specific effects of varying BMI levels on pregnancy outcomes.

Indeed, numerous studies included in our analysis utilized different cutoff values to define BMI categories, further complicating the interpretation of results. This variability in classification criteria not only hampers the comparability of findings across studies but also introduces potential inconsistencies in clinical decision-making. Without a standardized approach to BMI stratification, clinicians may face challenges in accurately assessing the impact of maternal BMI on FET outcomes and in tailoring treatment strategies. To address these challenges, future research should prioritize the implementation of harmonized classification criteria across BMI categories. Establishing universally accepted cutoff values for defining BMI categories would facilitate more precise clinical decision-making and enhance the comparability of findings across studies. Additionally, efforts to standardize outcome measures related to pregnancy outcomes in relation to maternal BMI would enhance the robustness of future research in this area. By addressing these challenges and promoting standardized approaches to BMI stratification, future studies can advance our understanding of the complex relationship between maternal BMI and FET outcomes, ultimately improving the quality of care for individuals undergoing ART.

## Future directions

The observed associations between maternal BMI and FET outcomes raise intriguing questions regarding underlying mechanisms and potential research directions. Mechanistic studies exploring the impact of BMI on endometrial receptivity, embryo quality, and implantation potential can provide deeper insights into the biological pathways mediating these associations. Furthermore, investigations into epigenetic modifications and gene expression patterns influenced by maternal BMI during embryonic development could identify additional factors that may contribute to pregnancy outcomes. Longitudinal studies investigating the effects of maternal BMI on long-term offspring health outcomes following FET are warranted to comprehensively assess intergenerational implications and inform clinical practice.

### Limitations and strengths of the study

It is imperative to recognize both the limitations and strengths inherent in our systematic review and meta-analysis. While the incorporation of a substantial number of studies reinforces the robustness of our findings, it’s crucial to acknowledge the potential biases and confounding factors introduced by the retrospective design of the included studies. Moreover, variations in study methodologies, including differences in sample sizes and BMI categorization criteria among the included studies, may affect the generalizability of our results.

Variability in sample sizes across studies underscores the need for cautious interpretation, as studies with larger sample sizes may have more substantial influence on the overall outcomes. Additionally, variations in cut-off values used to define BMI categories among the included studies pose challenges in synthesizing findings and may contribute to heterogeneity in results. However, despite these limitations, our study provides invaluable insights into the intricate association between maternal BMI and FET outcomes. By systematically synthesizing data from a diverse range of studies, our analysis offers a comprehensive overview of this complex relationship, laying a solid foundation for future research and the development of clinical practice guidelines. Moving forward, efforts to address these limitations, such as employing more rigorous study designs and standardizing methodologies for BMI categorization, will be essential to further enhance the reliability and applicability of findings in this critical area of research.

In conclusion, our systematic review and meta-analysis provide compelling evidence of the significant influence of maternal BMI on pregnancy outcomes following FET. These findings emphasize the need to consider maternal BMI as a critical determinant of reproductive success and advocate for personalized clinical approaches tailored to individual BMI profiles. By addressing gaps in existing literature and highlighting avenues for future research, our study contributes to advancing understanding of the complex interplay between maternal BMI and FET outcomes, with the ultimate goal of optimizing fertility treatment strategies and improving patient outcomes.

## Supporting information

S1 Table Search strategy. (DOCX)

S1 Fig Forest plot comparing live birth rate in underweight and normal-BMI women. (JPG)

S2 Fig Forest plot comparing live birth rate in overweight and normal-BMI women. (JPG)

S3 Fig Forest plot comparing live birth rate in obese and normal-BMI women. (JPG)

S4 Fig Forest plot comparing clinical pregnancy rate in underweight and normal-BMI women. (JPG)

S5 Fig Forest plot comparing clinical pregnancy rate in overweight and normal-BMI women. (JPG)

S6 Fig Forest plot comparing clinical pregnancy rate in obese and normal-BMI women. (JPG)

S1 File PRISMA 2020 Checklist. (DOCX)

S2 File List of excluded studies. (XLS)

S3 File Data extraction variables and all the eligible studies from which data extraction was done (20/4/2024 till 15/5/2024). (DOC)

## References

[1] Purewal S, Chapman SCE, van den Akker OBA. A systematic review and meta-analysis of lifestyle and body mass index predictors of successful assisted reproductive technologies. J Psychosom Obstet Gynaecol. 2019;40(1):2–18. doi: 10.1080/0167482X.2017.1403418 29172958

[2] Supramaniam PR, Mittal M, McVeigh E, Lim LN. The correlation between raised body mass index and assisted reproductive treatment outcomes: a systematic review and meta-analysis of the evidence. Reprod Health. 2018;15(1):34. doi: 10.1186/s12978-018-0481-z 29486787 PMC5830337

[3] D’Argenio V, Dittfeld L, Lazzeri P, Tomaiuolo R, Tasciotti E. Unraveling the Balance between Genes, Microbes, Lifestyle and the Environment to Improve Healthy Reproduction. Genes (Basel). 2021;12(4):605. doi: 10.3390/genes12040605 33924000 PMC8073673

[4] Dimitriadis E, Menkhorst E, Saito S, Kutteh WH, Brosens JJ. Recurrent pregnancy loss. Nat Rev Dis Primers. 2020;6(1):98. doi: 10.1038/s41572-020-00228-z 33303732

[5] Palomba S, Piltonen TT, Giudice LC. Endometrial function in women with polycystic ovary syndrome: a comprehensive review. Hum Reprod Update. 2021;27(3):584–618. doi: 10.1093/humupd/dmaa051 33302299

[6] Yang T, Zhao J, Liu F, Li Y. Lipid metabolism and endometrial receptivity. Hum Reprod Update. 2022;28(6):858–89. doi: 10.1093/humupd/dmac026 35639910

[7] Qiu M, Tao Y, Kuang Y, Wang Y. Effect of body mass index on pregnancy outcomes with the freeze-all strategy in women with polycystic ovarian syndrome. Fertil Steril. 2019;112(6):1172–9. doi: 10.1016/j.fertnstert.2019.08.009 31843094

[8] Rafael F, Rodrigues MD, Bellver J, Canelas-Pais M, Garrido N, Garcia-Velasco JA, et al. The combined effect of BMI and age on ART outcomes. Hum Reprod. 2023;38(5):886–94. doi: 10.1093/humrep/dead042 36928306

[9] Zheng Y, Dong X, Chen B, Dai J, Yang W, Ai J, et al. Body mass index is associated with miscarriage rate and perinatal outcomes in cycles with frozen-thawed single blastocyst transfer: a retrospective cohort study. BMC Pregnancy Childbirth. 2022;22(1):118. doi: 10.1186/s12884-022-04443-2 35148705 PMC8840631

[10] Gonzalez MB, Robker RL, Rose RD. Obesity and oocyte quality: significant implications for ART and emerging mechanistic insights. Biol Reprod. 2022;106(2):338–50. doi: 10.1093/biolre/ioab228 34918035

[11] Sermondade N, Huberlant S, Bourhis-Lefebvre V, Arbo E, Gallot V, Colombani M, et al. Female obesity is negatively associated with live birth rate following IVF: a systematic review and meta-analysis. Hum Reprod Update. 2019;25:439–51. doi: 10.1093/humupd/dmz011 30941397

[12] Brezina PR, Zhao Y. The ethical, legal, and social issues impacted by modern assisted reproductive technologies. Obstet Gynecol Int. 2012;2012:686253. doi: 10.1155/2012/686253 22272208 PMC3261493

[13] Halliday J, Wilson C, Hammarberg K, Doyle LW, Bruinsma F, McLachlan R, et al. Comparing indicators of health and development of singleton young adults conceived with and without assisted reproductive technology. Fertil Steril. 2014;101(4):1055–63. doi: 10.1016/j.fertnstert.2014.01.006 24559723

[14] Lawrenz B, Coughlan C, Melado L, Fatemi HM. The ART of frozen embryo transfer: back to nature!. Gynecol Endocrinol. 2020;36(6):479–83. doi: 10.1080/09513590.2020.1740918 32188299

[15] Wang B, Zhang J, Zhu Q, Yang X, Wang Y. Effects of different cycle regimens for frozen embryo transfer on perinatal outcomes of singletons. Hum Reprod. 2020;35(7):1612–22. doi: 10.1093/humrep/deaa093 32681726

[16] Lee JC, Badell ML, Kawwass JF. The impact of endometrial preparation for frozen embryo transfer on maternal and neonatal outcomes: a review. Reprod Biol Endocrinol. 2022;20(1):40. doi: 10.1186/s12958-021-00869-z 35227270 PMC8883648

[17] Yang J, He Y, Wu Y, Zhang D, Huang H. Association between abnormal body mass index and pregnancy outcomes in patients following frozen embryo transfer: a systematic review and meta-analysis. Reprod Biol Endocrinol. 2021;19(1):140. doi: 10.1186/s12958-021-00809-x 34503525 PMC8428102

[18] Rosalik K, Carson S, Pilgrim J, Luizzi J, Levy G, Heitmann R, et al. Effects of different frozen embryo transfer regimens on abnormalities of fetal weight: a systematic review and meta-analysis. Hum Reprod Update. 2021;28(1):1–14. doi: 10.1093/humupd/dmab037 34865039

[19] Zhang J, Liu H, Mao X, Chen Q, Fan Y, Xiao Y, et al. Effect of body mass index on pregnancy outcomes in a freeze-all policy: an analysis of 22,043 first autologous frozen-thawed embryo transfer cycles in China. BMC Med. 2019;17(1):114. doi: 10.1186/s12916-019-1354-1 31238940 PMC6593528

[20] Asserhøj LL, Mizrak I, Heldarskard GF, Clausen TD, Hoffmann ER, Greisen G, et al. Childhood BMI after ART with frozen embryo transfer. Hum Reprod. 2023;38(8):1578–89. doi: 10.1093/humrep/dead127 37349895

[21] Ben-Haroush A, Sirota I, Salman L, Son W-Y, Tulandi T, Holzer H, et al. The influence of body mass index on pregnancy outcome following single-embryo transfer. J Assist Reprod Genet. 2018;35(7):1295–300. doi: 10.1007/s10815-018-1186-5 29808381 PMC6063822

[22] Lin J, Guo H, Wang B, Zhu Q. Association of maternal pre-pregnancy body mass index with birth weight and preterm birth among singletons conceived after frozen-thawed embryo transfer. Reprod Biol Endocrinol. 2022;20(1):86. doi: 10.1186/s12958-022-00957-8 35689242 PMC9185967

[23] Higgins JPT. Commentary: Heterogeneity in meta-analysis should be expected and appropriately quantified. Int J Epidemiol. 2008;37(5):1158–60. doi: 10.1093/ije/dyn204 18832388

[24] Egger M, Davey Smith G, Schneider M, Minder C. Bias in meta-analysis detected by a simple, graphical test. BMJ. 1997;315(7109):629–34. doi: 10.1136/bmj.315.7109.629 9310563 PMC2127453

[25] Beshar I, Milki AA, Gardner RM, Zhang WY, Johal JK, Bavan B. Elevated body mass index in modified natural cycle frozen euploid embryo transfers is not associated with live birth rate. J Assist Reprod Genet. 2023;40: 1055–1062. doi: 10.1007/s10815-023-02787-y 37000344 PMC10239415

[26] Bakkensen JB, Strom D, Boots CE. Frozen embryo transfer outcomes decline with increasing female body mass index in female but not male factor infertility: analysis of 56,564 euploid blastocyst transfers. Fertil Steril. 2024;121(2):271–80. doi: 10.1016/j.fertnstert.2023.07.027 37549839

[27] Peterson A, Wu H, Kappy M, Kucherov A, Singh M, Lieman H, et al. Higher live birth rates are associated with a normal body mass index in preimplantation genetic testing for aneuploidy frozen embryo transfer cycles: a Society for Assisted Reproductive Technology Clinic Outcome Reporting System study. Fertil Steril. 2024;121(2):291–8. doi: 10.1016/j.fertnstert.2023.11.005 37952915

[28] Liu X, Shi J. Female obesity increases the risk of preterm birth of single frozen-thawed euploid embryos: a retrospective cohort study. Gynecol Endocrinol. 2024;40(1):2324995. doi: 10.1080/09513590.2024.2324995 38439198

[29] Fawarseh A, Atzmon Y, Aslih N, Bilgory A, Shalom-Paz E. Embryonic Development in Relation to Maternal Obesity Does Not Affect Pregnancy Outcomes in FET Cycles. Healthcare (Basel). 2022;10(4):703. doi: 10.3390/healthcare10040703 35455880 PMC9024931

[30] Shen X, Xie Y, Chen D, Guo W, Feng G, Jiang W, et al. Effect of Female and Male Body Mass Index on Cumulative Live Birth Rates in the Freeze-all Strategy. J Clin Endocrinol Metab. 2022;107(4):e1467–76. doi: 10.1210/clinem/dgab858 34850010

[31] Kidera N, Ishikawa T, Kawamura T, Miyasaka N. Maternal body mass index is not associated with assisted reproductive technology outcomes. Sci Rep. 2023;13(1):14817. doi: 10.1038/s41598-023-41780-4 37684397 PMC10491661

[32] Zheng L, Yang L, Guo Z, Yao N, Zhang S, Pu P. Obesity and its impact on female reproductive health: unraveling the connections. Front Endocrinol (Lausanne). 2024;14:1326546. doi: 10.3389/fendo.2023.1326546 38264286 PMC10803652

[33] Zeng Z, Li J, Wang X, Yi S, Bi Y, Mo D, et al. Influence of maternal obesity on embryonic vitrification injury and subsequent pregnancy outcomes: A retrospective cohort study. Heliyon. 2023;9(9):e20095. doi: 10.1016/j.heliyon.2023.e20095 37809804 PMC10559855

[34] Hu X, Yan E, Peng W, Zhou Y, Jin L, Qian K. Higher pre-pregnancy body mass index was associated with adverse pregnancy and perinatal outcomes in women with polycystic ovary syndrome after a freeze-all strategy: A historical cohort study. Acta Obstet Gynecol Scand. 2024;103: 884–896. doi: 10.1111/aogs.14771 38217337 PMC11019514

[35] Insogna IG, Lee MS, Reimers RM, Toth TL. Neutral effect of body mass index on implantation rate after frozen-thawed blastocyst transfer. Fertil Steril. 2017;108(5):770-776.e1. doi: 10.1016/j.fertnstert.2017.08.024 28985909

[36] Lin J, Huang J, Wang N, Kuang Y, Cai R. Effects of pre-pregnancy body mass index on pregnancy and perinatal outcomes in women with PCOS undergoing frozen embryo transfer. BMC Pregnancy Childbirth. 2019;19(1):487. doi: 10.1186/s12884-019-2611-1 31823750 PMC6902324

[37] Prost E, Reignier A, Leperlier F, Caillet P, Barrière P, Fréour T, et al. Female obesity does not impact live birth rate after frozen-thawed blastocyst transfer. Hum Reprod. 2020;35(4):859–65. doi: 10.1093/humrep/deaa010 32170315

[38] Tang S, Huang J, Lin J, Kuang Y. Adverse effects of pre-pregnancy maternal underweight on pregnancy and perinatal outcomes in a freeze-all policy. BMC Pregnancy Childbirth. 2021;21(1):32. doi: 10.1186/s12884-020-03509-3 33413207 PMC7791874

[39] Oliva M, Nazem TG, Lee JA, Copperman AB. Evaluating in vitro fertilization outcomes of patients with low body mass index following frozen-thawed embryo transfer. Int J Gynaecol Obstet. 2021;155(1):132–7. doi: 10.1002/ijgo.13570 33368250

[40] Fedorcsák P, Dale PO, Storeng R, Ertzeid G, Bjercke S, Oldereid N, et al. Impact of overweight and underweight on assisted reproduction treatment. Hum Reprod. 2004;19(11):2523–8. doi: 10.1093/humrep/deh485 15319380

[41] Cai J, Liu L, Zhang J, Qiu H, Jiang X, Li P, et al. Low body mass index compromises live birth rate in fresh transfer in vitro fertilization cycles: a retrospective study in a Chinese population. Fertil Steril. 2017;107: 422–9.e2. doi: 10.1016/j.fertnstert.2016.10.029 27887711

[42] Luke B, Brown MB, Stern JE, Missmer SA, Fujimoto VY, Leach R, et al. Female obesity adversely affects assisted reproductive technology (ART) pregnancy and live birth rates. Hum Reprod. 2011;26(1):245–52. doi: 10.1093/humrep/deq306 21071489

[43] Pasquali R, Gambineri A. Metabolic effects of obesity on reproduction. Reprod Biomed Online. 2006;12(5):542–51. doi: 10.1016/s1472-6483(10)61179-0 16790096

[44] Comninos AN, Jayasena CN, Dhillo WS. The relationship between gut and adipose hormones, and reproduction. Human Reproduction Update. 2013;20(2):153–74. doi: 10.1093/humupd/dmt033 24173881

[45] Mathew H, Castracane VD, Mantzoros C. Adipose tissue and reproductive health. Metabolism. 2018;86:18–32. doi: 10.1016/j.metabol.2017.11.006 29155136

[46] Broughton DE, Moley KH. Obesity and female infertility: potential mediators of obesity’s impact. Fertil Steril. 2017;107(4):840–7. doi: 10.1016/j.fertnstert.2017.01.017 28292619

[47] Campos DB, Palin M-F, Bordignon V, Murphy BD. The “beneficial” adipokines in reproduction and fertility. Int J Obes (Lond). 2008;32(2):223–31. doi: 10.1038/sj.ijo.0803719 17923861

[48] Dağ ZÖ, Dilbaz B. Impact of obesity on infertility in women. J Turk Ger Gynecol Assoc. 2015;16(2):111–7. doi: 10.5152/jtgga.2015.15232 26097395 PMC4456969

[49] Espinós JJ, Polo A, Sánchez-Hernández J, Bordas R, Pares P, Martínez O, et al. Weight decrease improves live birth rates in obese women undergoing IVF: a pilot study. Reprod Biomed Online. 2017;35(4):417–24. doi: 10.1016/j.rbmo.2017.06.019 28739335

[50] Kahn LG, Widen EM, Janevic T, Straka N, Liu X, Cirillo PM, et al. The Relation of Birth Weight and Adiposity Across the Life Course to Semen Quality in Middle Age. Epidemiology. 2019;30(Suppl 2):S17–27. doi: 10.1097/EDE.0000000000001070 31569149 PMC7055633

[51] Bellver J. In vitro fertilization in underweight women: focus on obstetric outcome. Fertil Steril. 2020;113: 323–4. doi: 10.1016/j.fertnstert.2019.10.009 31973901

[52] Romanski PA, Bortoletto P, Chung A, Magaoay B, Rosenwaks Z, Spandorfer SD. Reproductive and obstetric outcomes in mildly and significantly underweight women undergoing IVF. Reprod Biomed Online. 2021;42(2):366–74. doi: 10.1016/j.rbmo.2020.10.011 33243662

[53] Fontana R, Della Torre S. The Deep Correlation between Energy Metabolism and Reproduction: A View on the Effects of Nutrition for Women Fertility. Nutrients. 2016;8(2):87. doi: 10.3390/nu8020087 26875986 PMC4772050

[54] Velazquez MA, Fleming TP. Maternal diet, oocyte nutrition and metabolism and offspring health. Springer; 2013. pp. 329–351. Available: https://eprints.soton.ac.uk/369437/

[55] Marinelli S, Napoletano G, Straccamore M, Basile G. Female obesity and infertility: outcomes and regulatory guidance. Acta Biomed. 2022;93(4):e2022278. doi: 10.23750/abm.v93i4.13466 36043953 PMC9534231

[56] ESHRE Capri Workshop Group. Nutrition and reproduction in women. Hum Reprod Update. 2006;12(3):193–207. doi: 10.1093/humupd/dmk003 16449360

[57] Falcone V, Stopp T, Feichtinger M, Kiss H, Eppel W, Husslein PW, et al. Pregnancy after bariatric surgery: a narrative literature review and discussion of impact on pregnancy management and outcome. BMC Pregnancy Childbirth. 2018;18(1):507. doi: 10.1186/s12884-018-2124-3 30587161 PMC6307154

