# Supplementary material for: Impact of maternal body mass index on pregnancy outcomes following frozen embryo transfer: A systematic review and meta-analysis
Source: PLoS One. 2025 Mar 21;20(3):e0319012. doi: 10.1371/journal.pone.0319012 (PMC11927908; doi:10.1371/journal.pone.0319012)
Supplement: S1 Table — (DOCX) [file pone.0319012.s001.docx]

**S1 Table: Search strategy**

| **Query** | **Search Details** |
| --- | --- |
| ((((((obesity) OR (obese)) OR (overweight)) OR (body mass index)) AND (pregnancy)) AND (blastocyst transfer)) AND (frozen) | ("obeses"[All Fields] OR "obesity"[MeSH Terms] OR "obesity"[All Fields] OR "obese"[All Fields] OR "obesities"[All Fields] OR "obesity s"[All Fields] OR ("obeses"[All Fields] OR "obesity"[MeSH Terms] OR "obesity"[All Fields] OR "obese"[All Fields] OR "obesities"[All Fields] OR "obesity s"[All Fields]) OR ("overweight"[MeSH Terms] OR "overweight"[All Fields] OR "overweighted"[All Fields] OR "overweightness"[All Fields] OR "overweights"[All Fields]) OR ("body mass index"[MeSH Terms] OR ("body"[All Fields] AND "mass"[All Fields] AND "index"[All Fields]) OR "body mass index"[All Fields])) AND ("pregnancy"[MeSH Terms] OR "pregnancy"[All Fields] OR "pregnancies"[All Fields] OR "pregnancy s"[All Fields]) AND ("embryo transfer"[MeSH Terms] OR ("embryo"[All Fields] AND "transfer"[All Fields]) OR "embryo transfer"[All Fields] OR ("blastocyst"[All Fields] AND "transfer"[All Fields]) OR "blastocyst transfer"[All Fields]) AND ("freezing"[MeSH Terms] OR "freezing"[All Fields] OR "frozen"[All Fields]) |
| ((((((obesity) OR (obese)) OR (overweight)) OR (body mass index)) AND (birth rate)) AND (blastocyst transfer)) AND (frozen) | ("obeses"[All Fields] OR "obesity"[MeSH Terms] OR "obesity"[All Fields] OR "obese"[All Fields] OR "obesities"[All Fields] OR "obesity s"[All Fields] OR ("obeses"[All Fields] OR "obesity"[MeSH Terms] OR "obesity"[All Fields] OR "obese"[All Fields] OR "obesities"[All Fields] OR "obesity s"[All Fields]) OR ("overweight"[MeSH Terms] OR "overweight"[All Fields] OR "overweighted"[All Fields] OR "overweightness"[All Fields] OR "overweights"[All Fields]) OR ("body mass index"[MeSH Terms] OR ("body"[All Fields] AND "mass"[All Fields] AND "index"[All Fields]) OR "body mass index"[All Fields])) AND ("birth rate"[MeSH Terms] OR ("birth"[All Fields] AND "rate"[All Fields]) OR "birth rate"[All Fields]) AND ("embryo transfer"[MeSH Terms] OR ("embryo"[All Fields] AND "transfer"[All Fields]) OR "embryo transfer"[All Fields] OR ("blastocyst"[All Fields] AND "transfer"[All Fields]) OR "blastocyst transfer"[All Fields]) AND ("freezing"[MeSH Terms] OR "freezing"[All Fields] OR "frozen"[All Fields]) |
| (((((obesity) OR (obese)) OR (overweight)) OR (body mass index)) AND (birth rate)) AND (assisted reproductive technology) | ("obeses"[All Fields] OR "obesity"[MeSH Terms] OR "obesity"[All Fields] OR "obese"[All Fields] OR "obesities"[All Fields] OR "obesity s"[All Fields] OR ("obeses"[All Fields] OR "obesity"[MeSH Terms] OR "obesity"[All Fields] OR "obese"[All Fields] OR "obesities"[All Fields] OR "obesity s"[All Fields]) OR ("overweight"[MeSH Terms] OR "overweight"[All Fields] OR "overweighted"[All Fields] OR "overweightness"[All Fields] OR "overweights"[All Fields]) OR ("body mass index"[MeSH Terms] OR ("body"[All Fields] AND "mass"[All Fields] AND "index"[All Fields]) OR "body mass index"[All Fields])) AND ("birth rate"[MeSH Terms] OR ("birth"[All Fields] AND "rate"[All Fields]) OR "birth rate"[All Fields]) AND ("reproductive techniques, assisted"[MeSH Terms] OR ("reproductive"[All Fields] AND "techniques"[All Fields] AND "assisted"[All Fields]) OR "assisted reproductive techniques"[All Fields] OR ("assisted"[All Fields] AND "reproductive"[All Fields] AND "technology"[All Fields]) OR "assisted reproductive technology"[All Fields]) |
| (((((obesity) OR (obese)) OR (overweight)) OR (body mass index)) AND (birth rate)) AND (frozen embryo transfer) | ("obeses"[All Fields] OR "obesity"[MeSH Terms] OR "obesity"[All Fields] OR "obese"[All Fields] OR "obesities"[All Fields] OR "obesity s"[All Fields] OR ("obeses"[All Fields] OR "obesity"[MeSH Terms] OR "obesity"[All Fields] OR "obese"[All Fields] OR "obesities"[All Fields] OR "obesity s"[All Fields]) OR ("overweight"[MeSH Terms] OR "overweight"[All Fields] OR "overweighted"[All Fields] OR "overweightness"[All Fields] OR "overweights"[All Fields]) OR ("body mass index"[MeSH Terms] OR ("body"[All Fields] AND "mass"[All Fields] AND "index"[All Fields]) OR "body mass index"[All Fields])) AND ("birth rate"[MeSH Terms] OR ("birth"[All Fields] AND "rate"[All Fields]) OR "birth rate"[All Fields]) AND (("freezing"[MeSH Terms] OR "freezing"[All Fields] OR "frozen"[All Fields]) AND ("embryo transfer"[MeSH Terms] OR ("embryo"[All Fields] AND "transfer"[All Fields]) OR "embryo transfer"[All Fields])) |
| (((((obesity) OR (obese)) OR (overweight)) OR (body mass index)) AND (pregnancy)) AND (frozen embryo transfer) | ("obeses"[All Fields] OR "obesity"[MeSH Terms] OR "obesity"[All Fields] OR "obese"[All Fields] OR "obesities"[All Fields] OR "obesity s"[All Fields] OR ("obeses"[All Fields] OR "obesity"[MeSH Terms] OR "obesity"[All Fields] OR "obese"[All Fields] OR "obesities"[All Fields] OR "obesity s"[All Fields]) OR ("overweight"[MeSH Terms] OR "overweight"[All Fields] OR "overweighted"[All Fields] OR "overweightness"[All Fields] OR "overweights"[All Fields]) OR ("body mass index"[MeSH Terms] OR ("body"[All Fields] AND "mass"[All Fields] AND "index"[All Fields]) OR "body mass index"[All Fields])) AND ("pregnancy"[MeSH Terms] OR "pregnancy"[All Fields] OR "pregnancies"[All Fields] OR "pregnancy s"[All Fields]) AND (("freezing"[MeSH Terms] OR "freezing"[All Fields] OR "frozen"[All Fields]) AND ("embryo transfer"[MeSH Terms] OR ("embryo"[All Fields] AND "transfer"[All Fields]) OR "embryo transfer"[All Fields])) |
