# Supplementary figures and images for: Impact of maternal body mass index on pregnancy outcomes following frozen embryo transfer: A systematic review and meta-analysis
Source: PLoS One. 2025 Mar 21;20(3):e0319012. doi: 10.1371/journal.pone.0319012 (PMC11927908; doi:10.1371/journal.pone.0319012)

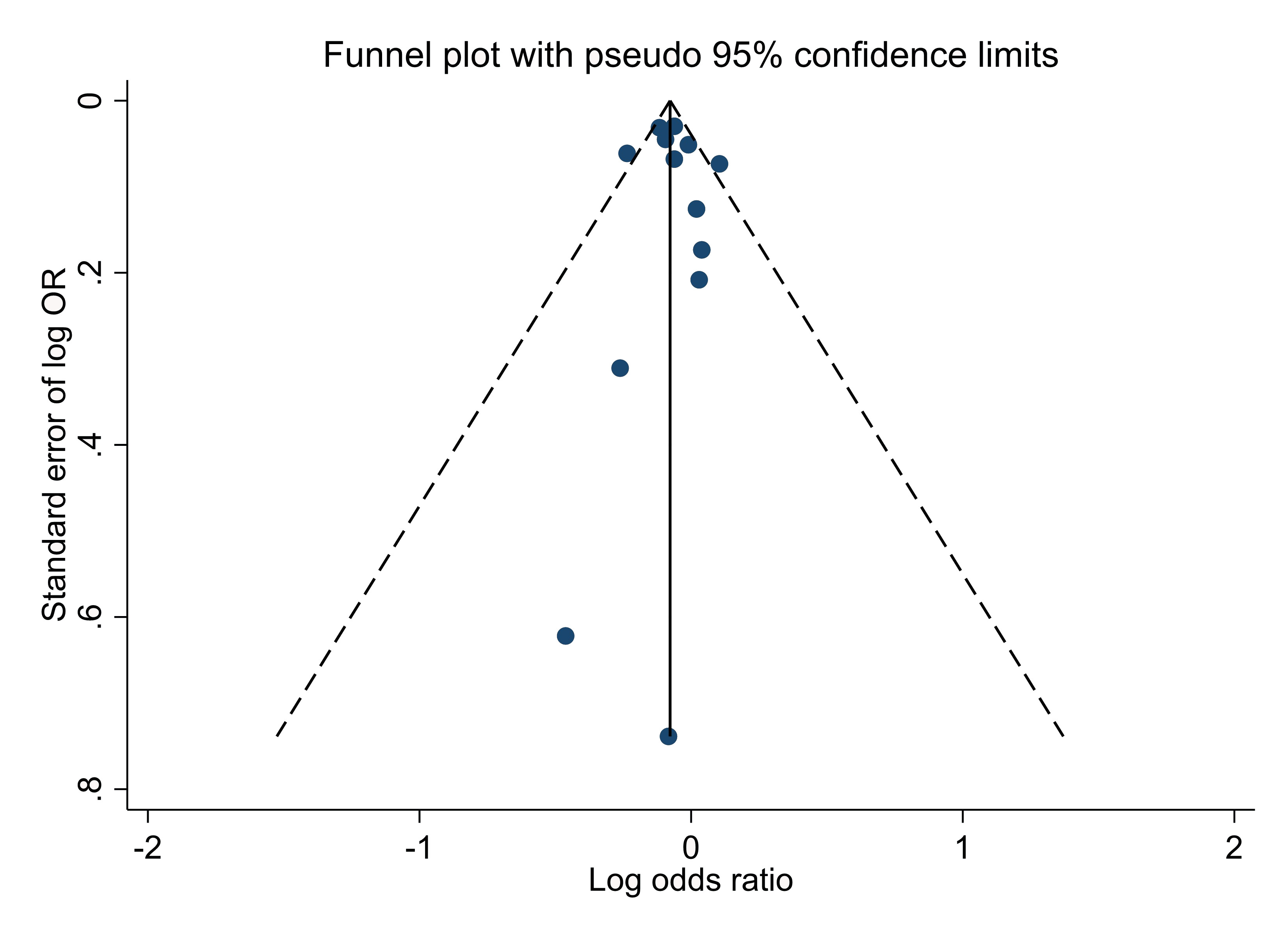

Supplement: S1 Fig — (JPG) [file pone.0319012.s002.jpg]

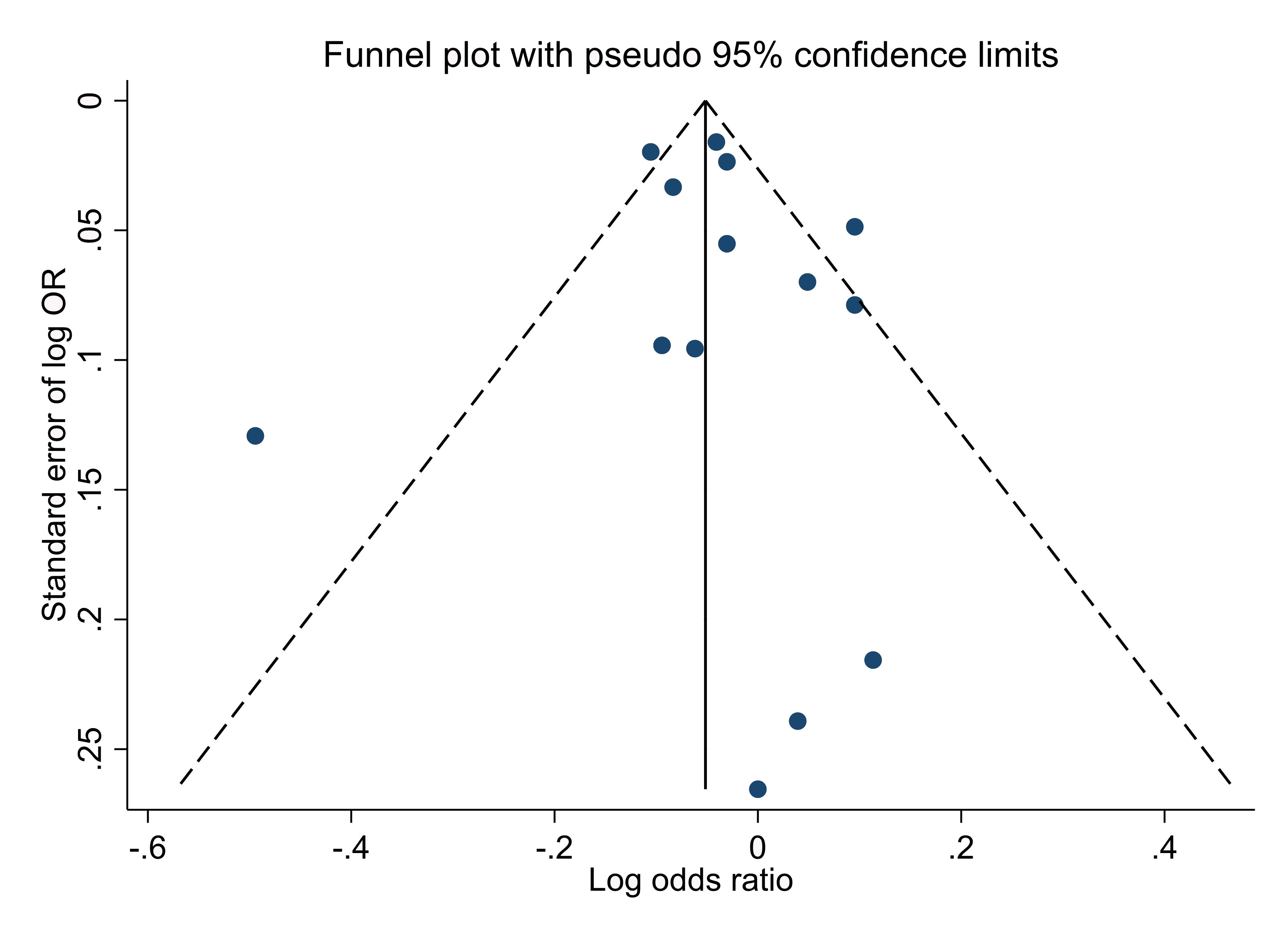

Supplement: S2 Fig — (JPG) [file pone.0319012.s003.jpg]

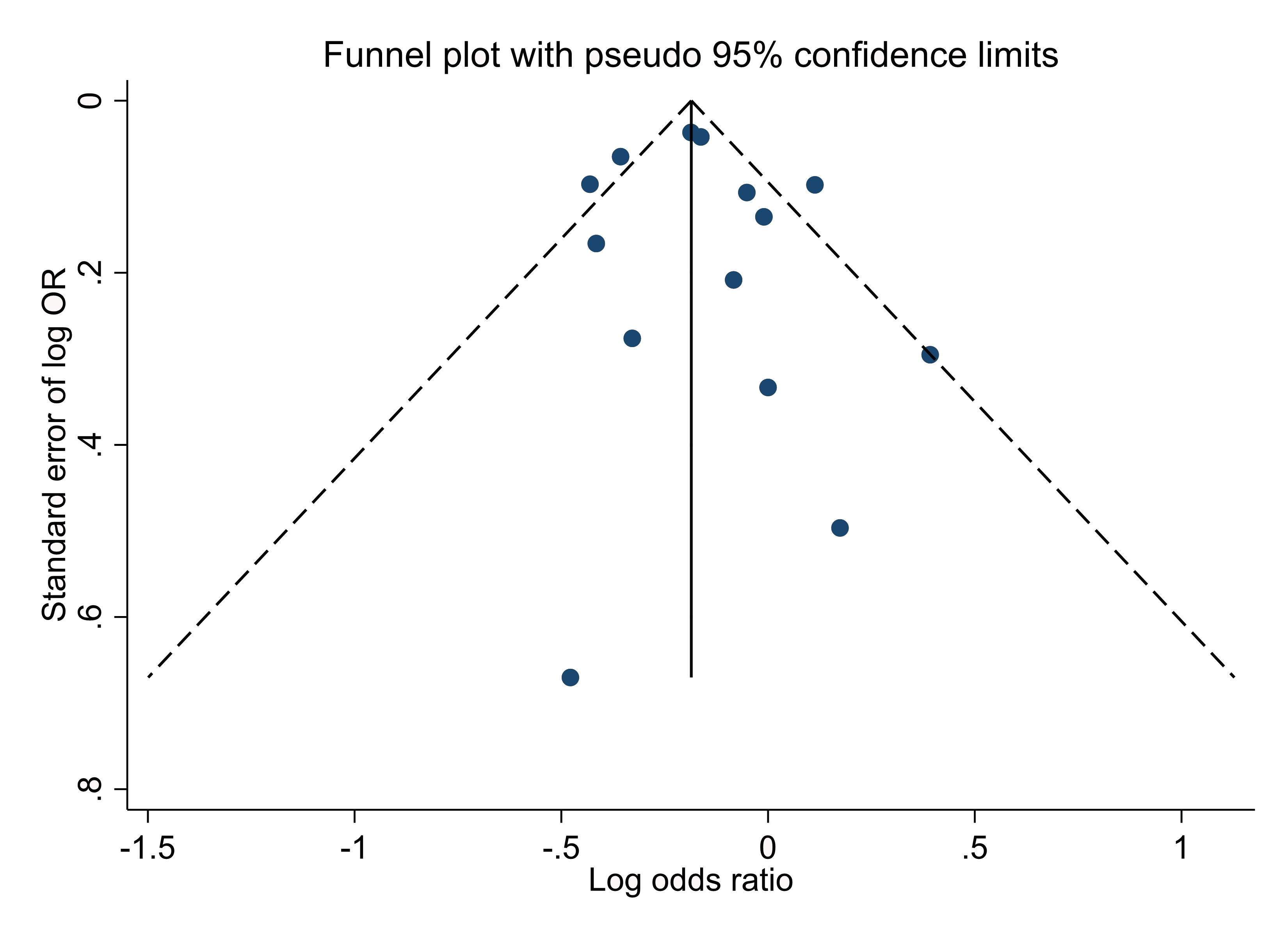

Supplement: S3 Fig — (JPG) [file pone.0319012.s004.jpg]

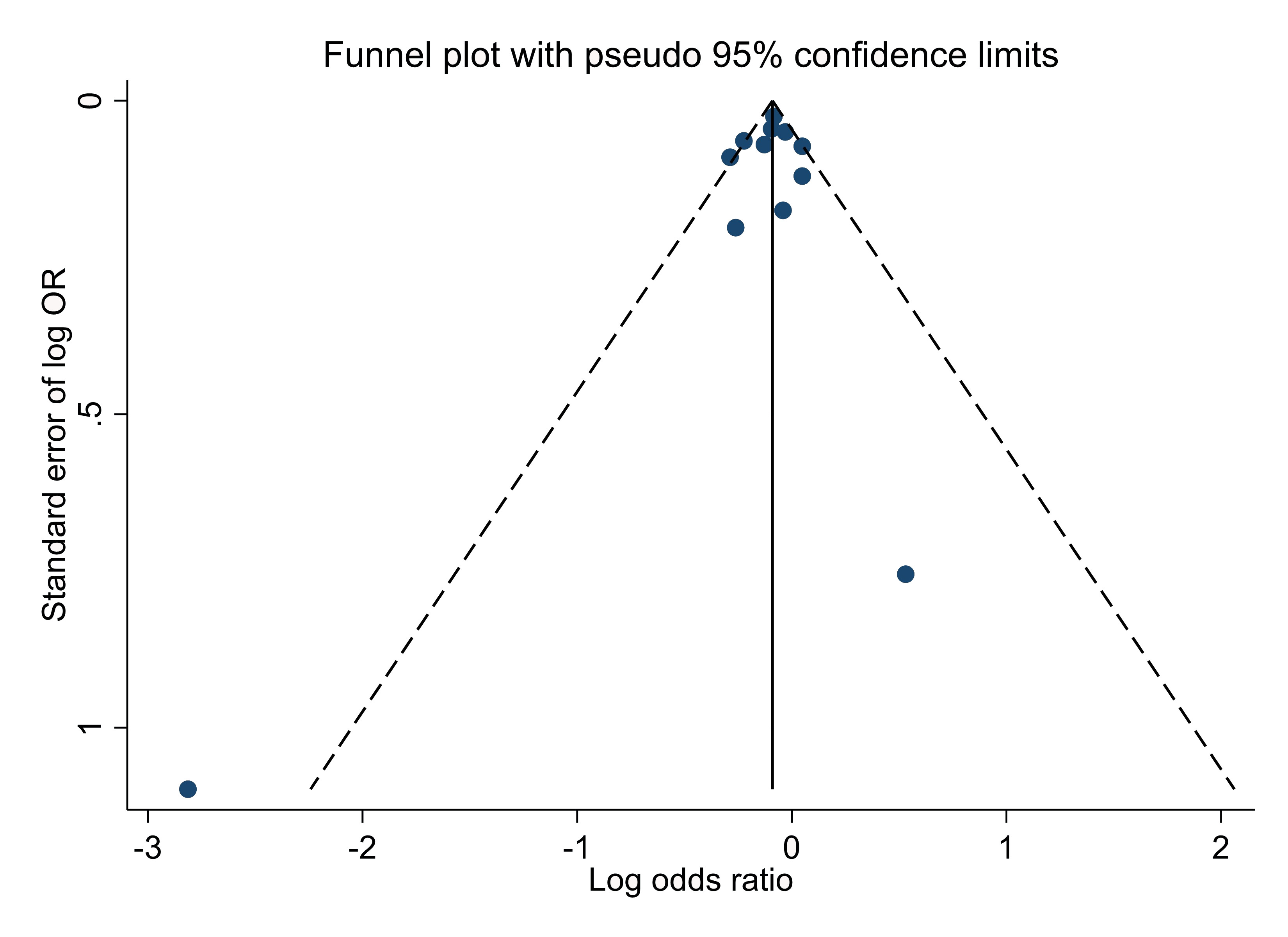

Supplement: S4 Fig — (JPG) [file pone.0319012.s005.jpg]

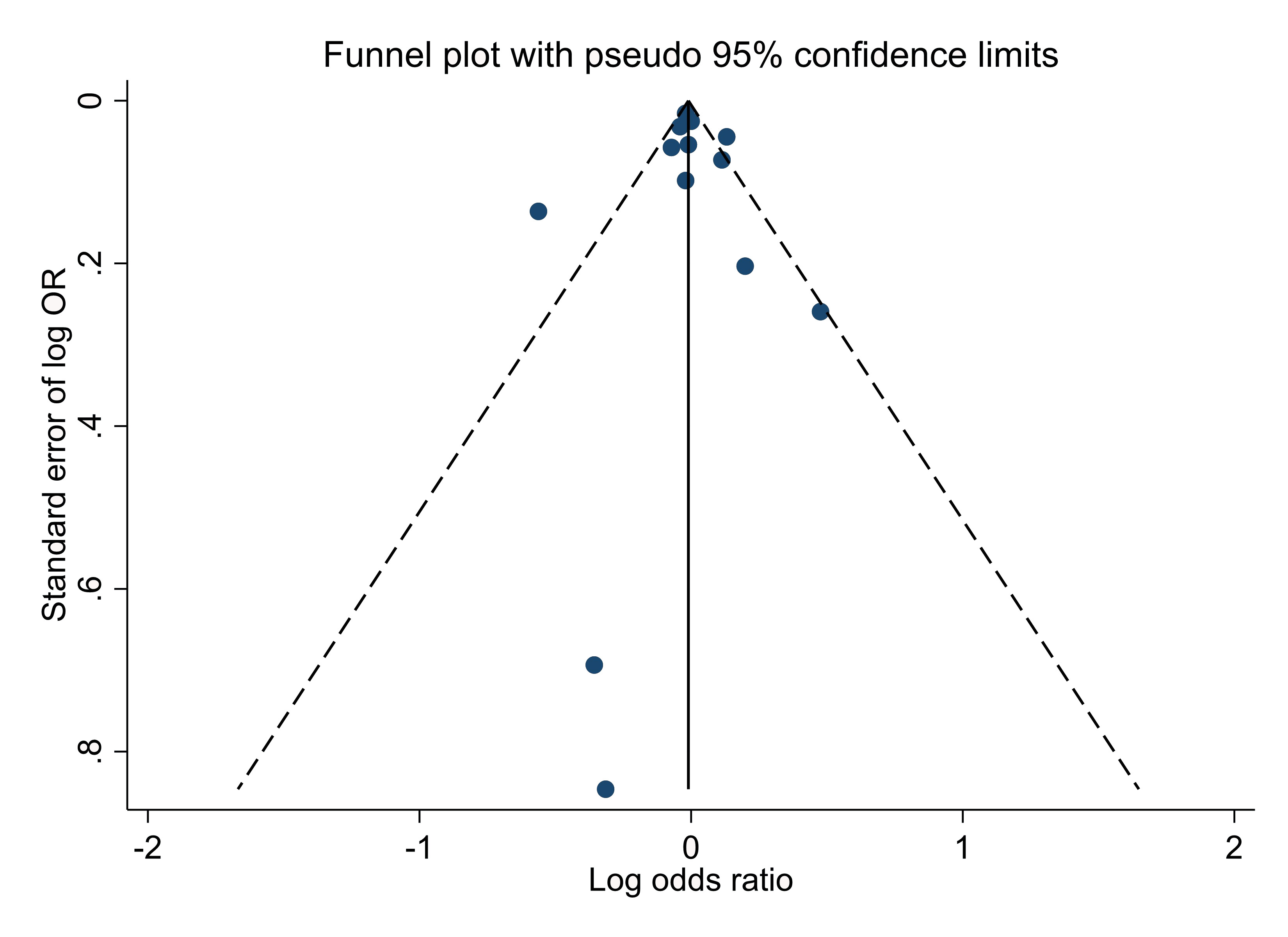

Supplement: S5 Fig — (JPG) [file pone.0319012.s006.jpg]

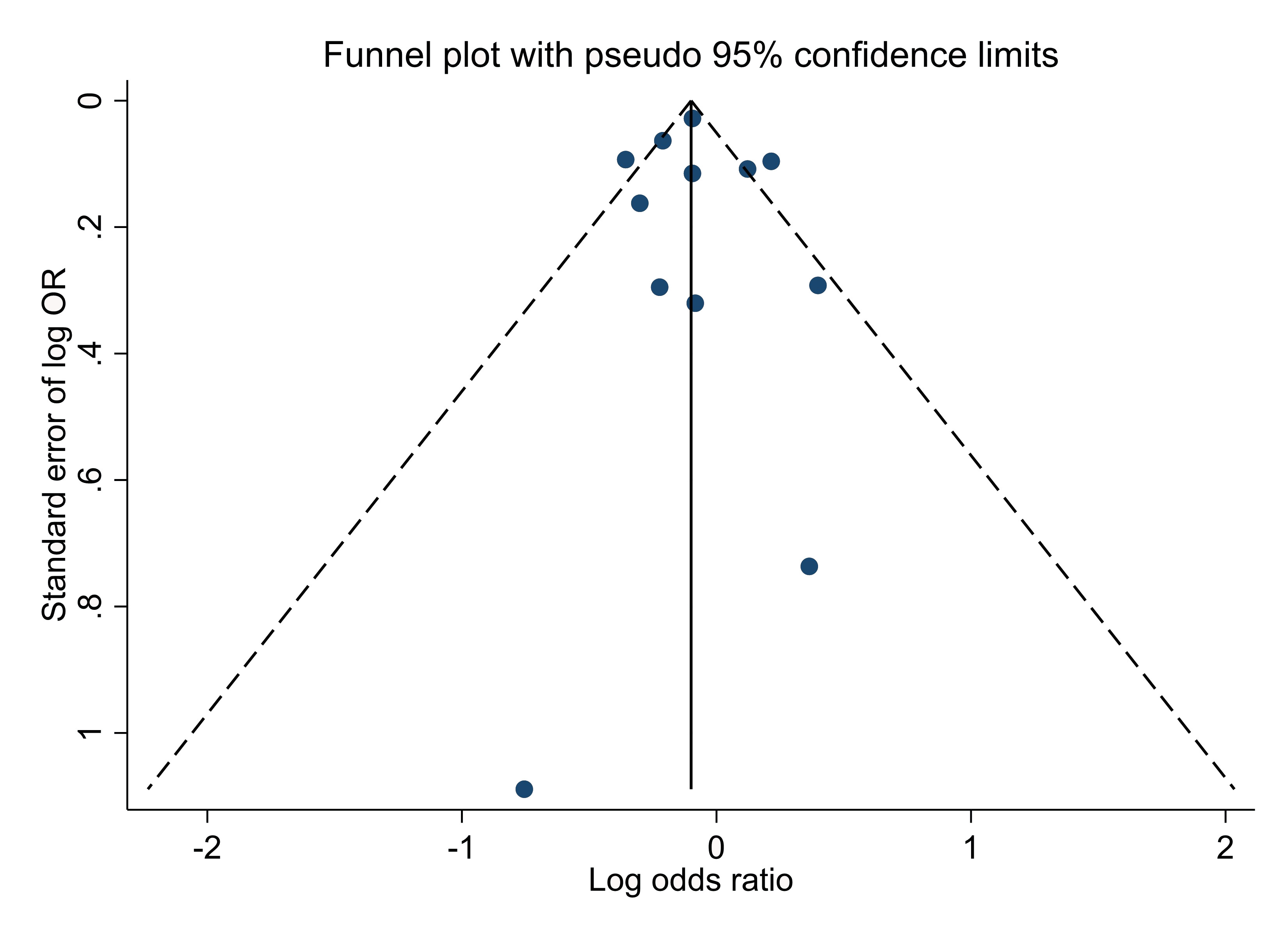

Supplement: S6 Fig — (JPG) [file pone.0319012.s007.jpg]
