# Supplementary material for: Impact of maternal body mass index on pregnancy outcomes following frozen embryo transfer: A systematic review and meta-analysis
Source: PLoS One. 2025 Mar 21;20(3):e0319012. doi: 10.1371/journal.pone.0319012 (PMC11927908; doi:10.1371/journal.pone.0319012)
Supplement: S3 File — (DOC) [file pone.0319012.s010.doc]

**S3 File. Data extraction variables and all the eligible studies from which data extraction was done (20/4/2024 till 15/5/2024)**

| **References; study design** | **Data extractors** | **Country** | **Groups** | **Sample size** | **Mean age (years)** | **BMI definition (kg/m^2^)** | **Infertility duration (years)** | **Adjustment done for** |
| --- | --- | --- | --- | --- | --- | --- | --- | --- |
| Beshar et al. (2023)  RC | Chucheng Tang and Fengming Tu | USA | Normal  Overweight  Obese | 229  128  68 | 22  26.3  33.0 | 18.5-24.9  25-30  >30 | NR | Age at transfer, nulliparity, embryo grade,  race/ethnicity, endometrial thickness on day of hCG trigger, and diagnosis of unexplained infertility |
| Bakkensen et al. (2024)  RC | Chucheng Tang and Fengming Tu | USA | Underweight  Normal  Overweight  Obese | 1612  31666  13419  9867 | 34.3±4.4  35.2±4.1  35.4±4.1  35.6±4.1 | <18.5  18.5-24.9  25-29.9  ≥30 | NR | Age at transfer, race and ethnicity, prior pregnancy loss, current smoking, indication for preimplantation genetic testing, and endometrial thickness |
| Peterson et al. (2024)  RC | Chucheng Tang and Fengming Tu | USA | Underweight  Normal  Overweight  Obese | 1734  33126  13068  7960 | 34.7±4.0  35.1±4.2  35.3±4.2  35.6±4.0 | <18.5  18.5-24.9  25-29.9  >30 | NR | Age, cycle order, race, male factor infertility, and female factor infertility |
| Liu and Shi (2024)  RC | Chucheng Tang and Fengming Tu | China | Underweight  Normal  Overweight  Obese | 81  637  108  25 | 30.6±4.0  31.1±3.4  31.7±4  30±4.2 | <18.5  18.5-24.9  25-30  >30 | 2.2±2.1  2.0±2.1  2.8±2.5  3.0±2.0 | Infertility duration, endometrial thickness, infer­tility type (primary infertility vs secondary infertility), protocol in fresh cycle (agonist, antagonist, other), biopsied blastocysts, no result embryos |
| Fawarseh et al. (2022)  RC | Chucheng Tang and Fengming Tu | Israel | Underweight  Normal  Overweight  Obese | 43  286  154  158 | 33.3±7.2  34.9±6.2  35.9±7.4  36.1±5.2 | <18.5  18.5-24.9  25-30  >30 | NR | Maternal age, endometrial thickness, and KID scores (reflecting embryo quality) |
| Shen et al. (2022)  RC | Chucheng Tang and Fengming Tu | China | Underweight  Normal  Overweight  Obese | 2422  13845  6037  1178 | 31±4.3  32.3±4.8  33.1±5.2  31.9±4.9 | <18.5  18.5-<23  23-<27.5  ≥27.5 | 3.1±2.6  3.2±2.9  3.5±3.3  3.7±3.3 | Adjustment for confounders done; however, variables adjusted were not mentioned |
| Kidera et al. (2023)  RC | Chucheng Tang and Fengming Tu | Japan | Underweight  Normal  Overweight  Obese | 943  3814  935  330 | 38  39  40  41 | <18.5  18.5-22.5  22.5 to 25  >25 | NR | Propensity score matched |
| Zheng et al. (2022)  RC | Chucheng Tang and Fengming Tu | China | Underweight  Normal  Overweight  Obese | 1127  6925  1810  390 | 30.2±3.9  31.9±4.5  32.8±5.2  31.7±4.6 | <18.5  18.5-24  24 to 28  ≥28 | 3.0±1.9  3.2±2.3  3.6±2.6  3.8±2.4 | Maternal age, type of infertility, IVF indications,  antral follicle count (AFC), endometrial thickness,  type of endometrial preparation, expansion stage, inner cell mass, and trophectoderm |
| Zeng et al. (2023)  RC | Chucheng Tang and Fengming Tu | China | Underweight  Normal  Overweight  Obese | 2136  11723  2622  495 | NR | <18.5  18.5-23.9  24 to 27.9  ≥28 | NR | Maternal age, paternal age, causes of infertility, protocol, number of high-quality embryos and D3/D5 transferred embryos |
| Hu et al. (2024)  RC | Chucheng Tang and Fengming Tu | China | Underweight  Normal  Overweight  Obese | 137  1130  339  61 | 28  29  29  29 | <18.5  18.5-<25  25 to <30  ≥30 | 3  3  3  4 | Maternal age, number of embryos transferred, stage of embryo development, endometrial preparation protocol, fertilization method, cause of  infertility, endometrial thickness, and number of oocytes retrieved |
| Insogna et al. (2017)  RC | Chucheng Tang and Fengming Tu | USA | Underweight  Normal  Overweight  Obese | 8  288  106  59 | 34.8±3.2  35.4±4.2  36.6±5.0  36.6±3.9 | <18.5  18.5-24.9  25 to 29.9  ≥30 | NR | Cohort score (marker of embryo quality), uterine cause of infertility, mock transfer score, maternal age, transfer of more than one embryo, diminished ovarian reserve, and male factor infertility |
| Zhang et al. (2019)  RC | Chucheng Tang and Fengming Tu | China | Underweight  Normal  Overweight  Obese | 2527  13224  5079  1213 | 30.5  31.3  31.6  31.1 | <18.5  18.5-<23  23 to 27.5  >27.5 | 3.2±2.3  3.3±2.7  3.6±2.9  4.1±2.9 | Maternal age, infertility duration, gravidity, parity, main cause of infertility, number of OPU prior to FET, year of treatment, number of embryos transferred, and embryo developmental stage at transfer |
| Lin et al. (2019)  RC | Chucheng Tang and Fengming Tu | China | Underweight  Normal  Obese | 972  480  228 | 32.8±3.4  33.1±3.8  33.3±3.6 | 18.5-24.9  25 to 29.9  ≥30 | 3.8±2.7  42.9  4.2±2.9 | Maternal age, infertility duration, duration of cryopreservation, endometrial thickness, embryo quality, means of preparing the  endometrium, number of embryos transferred as well as embryo developmental stage |
| Prost et al. (2020)  RC | Chucheng Tang and Fengming Tu | France | Normal  Obese | 799  159 | 32.9±4.1  32.9±4.8 | 18.5- 24.9  ≥30 | NR | Maternal age, smoking status, serum AMH, endometrium thickness, parity, infertility cause, double blastocyst transfer |
| Qiu et al. (2019)  RC | Chucheng Tang and Fengming Tu | China | Underweight  Normal  Overweight  Obese | 184  1911  780  204 | 28.9±3.2  29.9±3.4  30.5±3.9  30±3.7 | <18.5  18.5-24.9  25 to 29.9  ≥30 | 3.1±2.0  3.5±2.6  3.9±2.8  4.2±2.9 | Female age, duration of infertility, gravidity, parity, history of preterm delivery, indication combined with PCOS, previous IVF failures, AFC, fertilization methods,  embryo stage at transfer, endometrial thickness on ET day, endometrial preparation, and the number of embryos transferred |
| Tang et al. (2021)  RC | Chucheng Tang and Fengming Tu | China | Underweight  Normal  Overweight | 1315  6230  1210 | 33.6  33.8  33.7 | <18.5  18.5-24.9  ≥25 | NR | Age of embryo transfer, age of oocyte retrieval, infertility duration, endometrial thickness,  embryo quality, number of embryos transferred, and embryo developmental stage |
| Oliva et al. (2021)  RC | Chucheng Tang and Fengming Tu | USA | Underweight  Normal | 314  4420 | NR | <18.5  18.5-24.9 | NR | Maternal age, markers of ovarian reserve, total  gonadotropin dose, stimulation type, trigger type, estradiol and progesterone  at the time of surge, number of embryos transferred, day of  embryo transfer, endometrial type, and morphologic grade |

RC, retrospective cohort; NR, not reported; BMI, body mass index; PCOS, polycystic ovary syndrome
